# An in vitro investigation to understand the synergistic role of MMPs-1 and 9 on articular cartilage biomechanical properties

**DOI:** 10.1038/s41598-021-93744-1

**Published:** 2021-07-13

**Authors:** Allison Mixon, Andrew Savage, Ahmed Suparno Bahar-Moni, Malek Adouni, Tanvir Faisal

**Affiliations:** 1 Department of Mechanical Engineering, University of Louisiana at Lafayette, Lafayette, LA 70503 USA; 2 Department of Biology, University of Louisiana at Lafayette, Lafayette, LA 70503 USA; 3 Department of Orthopaedics, Advanced Medical and Dental Institute, Universiti Sains Malaysia, Bertam, 13200 Kepala Batas, Penang Malaysia; 4 Department of Mechanical Engineering, Australian College of Kuwait, P.O. Box 1411, East Meshrif, Kuwait

**Keywords:** Biomedical engineering, Cartilage

## Abstract

Matrix metalloproteinases (MMPs) play a crucial role in enzymatically digesting cartilage extracellular matrix (ECM) components, resulting in degraded cartilage with altered mechanical loading capacity. Overexpression of MMPs is often caused by trauma, physiologic conditions and by disease. To understand the synergistic impact MMPs have on cartilage biomechanical properties, MMPs from two subfamilies: collagenase (MMP-1) and gelatinase (MMP-9) were investigated in this study. Three different ratios of MMP-1 (c) and MMP-9 (g), c1:g1, c3:g1 and c1:g3 were considered to develop a degradation model. Thirty samples, harvested from bovine femoral condyles, were treated in groups of 10 with one concentration of enzyme mixture. Each sample was tested in a healthy state prior to introducing degradative enzymes to establish a baseline. Samples were subjected to indentation loading up to 20% bulk strain. Both control and treated samples were mechanically and histologically assessed to determine the impact of degradation. Young’s modulus and peak load of the tissue under indentation were compared between the control and degraded cartilage explants. Cartilage degraded with the c3:g1 enzyme concentration resulted in maximum 33% reduction in stiffness and peak load compared to the other two concentrations. The abundance of collagenase is more responsible for cartilage degradation and reduced mechanical integrity.

## Introduction

A distinctive feature of arthritic disease is cartilage extracellular matrix (ECM) degradation, which is often orchestrated by matrix metalloproteinases (MMPs) as well as a disintegrin and metalloproteinase with thrombospondin type I motifs (ADAMTSs). Epidemiological studies show that either disease^1^ or acute trauma^2^ to the articular cartilage triggers pro-inflammatory cytokines such as IL-1 beta, and TNF-alpha^3^, which stimulate the production of MMPs and disrupt their healthy balance. While MMPs are important regulators for tissue homeostasis, unregulated MMP activity turns destructive and is closely associated with arthritic disease pathways. MMPs degrade two principal macromolecules of cartilage ECM— proteoglycan (PG) aggregates and type II collagen fibrils^4^, beginning at the articular surface^5^. The fibrils are the major load-bearing components that provide tensile strength and aid to restrict PG swelling, and thereby endowing cartilage with its compressive stiffness^6,7^. PG degradation is thought to be an early and reversible process, whereas the breakdown (degradation) of the collagen network is believed to be an irreversible loss of tensile properties and structural integrity^8^. The collagen damage inflicted by degradative enzymes upon the articular cartilage surface during early-stage osteoarthritis (OA) has long been recognized as one of the earliest stages of the disease process^9–11^ and is believed to be the point of no return for disease progression^11^.

The breakdown of the ECM by different MMPs is closely related to degenerative joint diseases (such as OA) and a major cause of joint dysfunction^12–14^. MMPs-1, 2, 9, 13 and others have been found in elevated levels within the synovial fluid of the pathways to cartilage destruction^15,16^. Traditionally known as collagenases and gelatinases, these enzymes act in two steps, first collagenases (MMPs-1, 8 or 13) cleave and bind triple helical collagen molecules and denature the collagen fibrils^17–19^, and thereafter, gelatinases (MMPs-2, 9) digest the denatured fibers^18,20,21^. In the present work, two key members of MMP family, MMP-1 (interstitial collagenase) and MMP-9 (gelatinase B) were primarily considered representatives of two protease classes. Other collagenases, such as MMP-13 have often been studied^22–24^ as the major catabolic effector in OA and have been perceived to be more active than MMP-1 on type II collagen. However, the level of MMP-1 expression is often tenfold higher than that of MMP-13 expression^25^, suggesting that the sheer amount of MMP-1 can overcome its comparative lack of efficiency in degrading the load-bearing type II collagen fibers. MMP-9, which acts as collagenase and gelatinase^26^, is the most complex member of MMP families in terms of proteolytic activity and may individually degrade non-collagen matrix components. Overexpression of MMPs results in an imbalance between the activity of MMPs that becomes pathogenic to cartilage. Therefore, the current study aims to understand the role of relative concentration of two classes of MMPs, MMP-1 and MMP-9, when they act in synergy on cartilage degradation mechanism.

The synergistic effect of a weakening cartilage infrastructure (due to degradation) and mechanical stresses on cartilage and joints accelerate the characteristic wearing away of the articular cartilage. To understand the correlation between the biochemical changes and mechanical loading, a limited number of in vivo studies based on contact forces were conducted on OA patients’ knee joints^27–29^. These studies failed to address the kinematic changes in degraded cartilage associated with early-stage OA^30^. Clinical cartilage testing is conducted utilizing slow strain rate indentation tests (post-mortem) as a method of evaluating degenerative changes observed in cartilage. Alternatively, transient indentation testing is of interest for in vitro studies to measure compressive stiffness along with mild cartilage degeneration^31,32^. The relationship between degraded matrix constituents and cartilage’s mechanical integrity was discreetly investigated in vitro through enzymatic degradation^33–37^. Indeed, in vitro degradation models containing a variety of enzymes have been used in the past to simulate ECM degradation in the context of osteoarthritis^34,36,37^, but these models are limited. Furthermore, the catabolic effect of combined MMP-1 and MMP-9 on the structural integrity (stiffness and/or load-bearing ability) of cartilage tissue has not been studied explicitly. Hence, the current in vitro study is designed to investigate the macro-mechanical properties of cartilage treated with different concentrations of MMP-1 and MMP-9 in combination to unfold the mutual effect of MMPs on cartilage biomechanics.

Accordingly, in this study, an in vitro cartilage degradation model with ECM degeneration at the articular surface treated with three different concentration ratios of MMP-1(c) to MMP-9 (g) as 1:3, 1:1 and 3:1, denoted by c1:g3, c1:g1, and c3:g1, respectively, was developed to explore the comparative influence of the relative concentration of collagenase and gelatinase. Mechanical integrity such as the intrinsic, equilibrium Young’s modulus $\left( E \right)$ and the peak load at 20% bulk strain of the cartilage tissue was measured and compared under indentation loading for both intact and enzymatically degraded cartilage explants. This study enables us to understand the pathomechanics of cartilage tissue, which may eventually pave the development of a targeted tissue inhibitors of metalloproteinases (TIMP) and therapeutic interventions.

## Results

### Mechanical characterization

**Figure 1:**
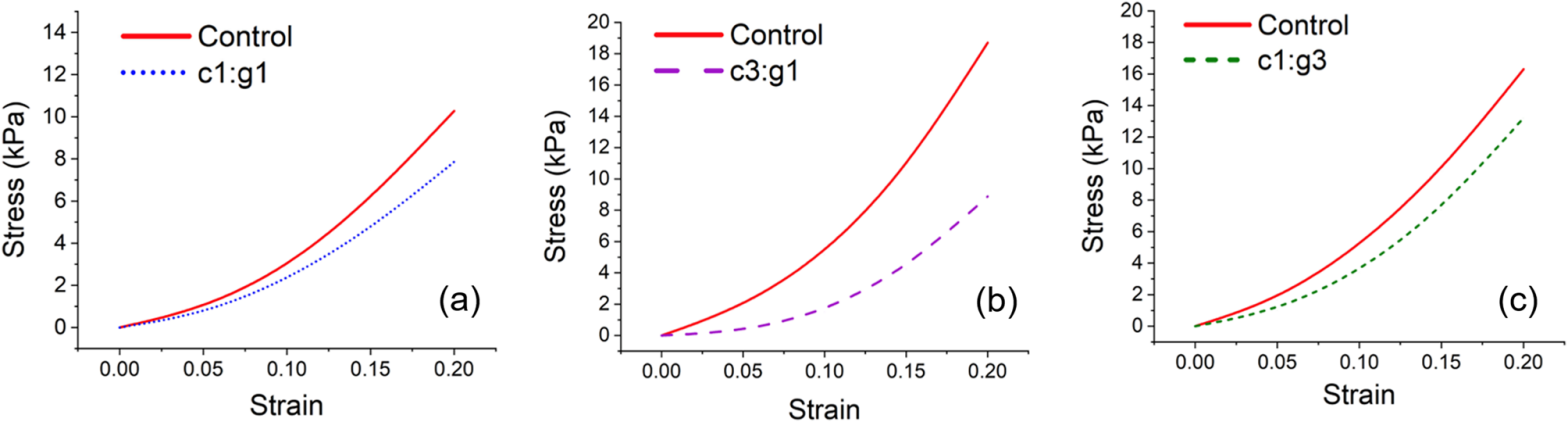
Stress–strain analyses of healthy and degraded cartilage samples before and after treatment with enzyme concentrations of c1:g1 (**a**), c3:g1 (**b**), and c1:g3 (**c**).

Indentation testing has shown its potential for assessing mechanical properties of articular cartilage^31,32,38–40^ and is adopted in this study to characterize the mechanical integrity of enzymatically degraded cartilage. All the samples, irrespective of control or treatment groups, were subjected to 20% bulk strain. The stress–strain analyses show a declining load-bearing ability of cartilage explants with increasing strain (Fig. 1) for each treated group as compared with their respective control group. The analyses show that the decrease in compressive stress for c3:g1 is significant compared with the other two concentrations of enzymes (c1:g1 and c1:g3). Untreated cartilage samples belonging to the c3:g1 group exhibited compressive stress as high as 19 kPa under 20% strain; after treatment the same samples reached maximum stress at only 9 kPa for the same level of strain. The stress at 20% strain between the control and treated group are ~ 17 kPa and ~ 13 kPa, and ~ 11 kPa and ~ 7.8 kPa for c1:g1 and c1:g3, respectively.

**Figure 2:**
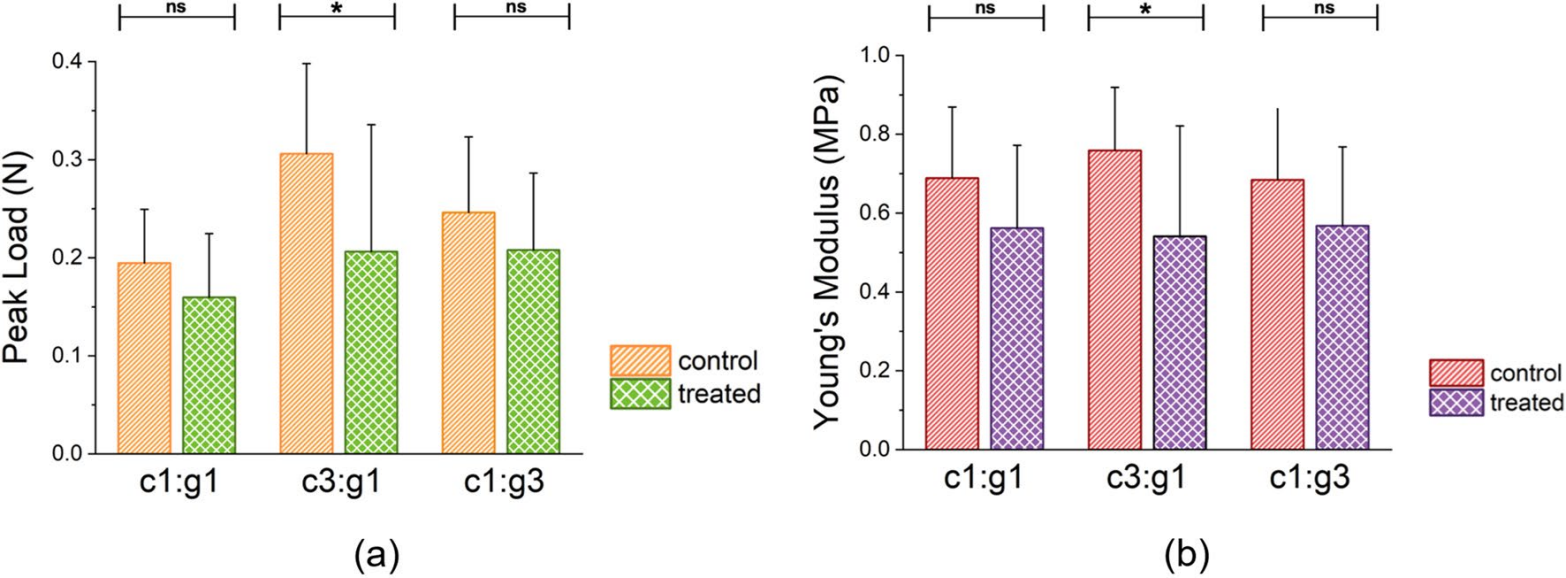
(**a**) Average peak load $\left( {mean \pm SD} \right)$ required in indentation per treatment group and the associated control group at 20% of applied bulk strain. (**b**) Average Young’s modulus $\left( {mean \pm SD} \right)$ for each treatment group and the associated control group.

Figure 2a shows the changes in peak load (mean ± SD) for the three treated groups compared with their control data. Out of the three treatment groups, cartilage specimens enzymatically degraded with the c3:g1 concentration of mixture experienced a statistically significant decrease in peak load compared to their control baseline data. The c1:g1, c1:g3, and c3:g1 treatment groups resulted in an average of 19%, 16%, and 33% reduction in the peak load required to strain the samples 20% of the original cartilage thickness in their degraded state (Fig. 2a). Prior to enzymatic digestion by MMPs-1 and 9, the statistically significant treatment group (c3: g1) experienced an average peak load of 0.31 N at 20% of strain. Following treatment, the same group of samples only required an average peak load of 0.21 N to reach the same level of compressive strain tested at the same strain rate.

Figure 2b shows the average variation of Young’s modulus among the different treatment groups. The elastic modulus data extracted from the indentation tests also resulted in a statistically significant decrease for the c3:g1 treatment group when compared to its control baseline. While the degradative changes to the c1:g1 and c1:g3 groups did not reveal statistical significance $\left( {p < 0.05} \right)$ in their biomechanical properties, the cartilage response for all three groups followed a decreasing trend when tested after enzymatic degradation. A similar behavior pattern to the peak load data was observed in the Young’s modulus where the c1:g1, c1:g3, and c3:g1 treatment groups revealed a 19%, 17%, and 33% decrease in elastic modulus, respectively (Fig. 2b).

**Figure 3:**
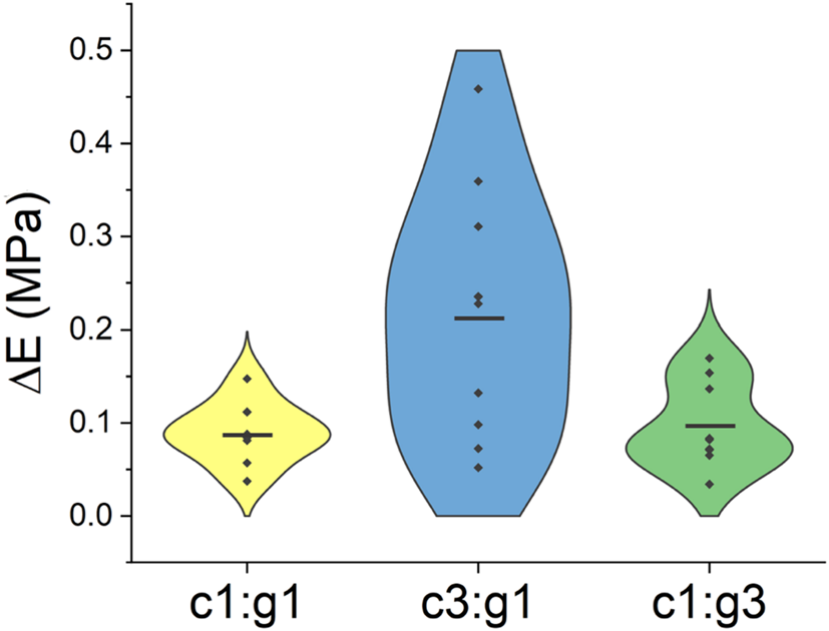
Distributions of changes in elastic modulus $\left( {\Delta E} \right)$ of degraded cartilage for c1:g1, c3:g1, and c1:g3 treatments groups. The mean of each group is represented by a straight line. Individual data points within the distribution are shown.

The violin plot in Fig. 3 shows the distributions of differences in elastic modulus $\left( {\Delta E} \right)$ between healthy and degraded cartilage for the three combinations of enzyme (MMPs) mixtures as a continuous approximation of the probability density function, computed using kernel density estimation (KDE). The width of each curve corresponds with the approximate frequency of data points in each region. Wider sections of the violin plot represent a higher probability, and the skinnier sections represent a lower probability. Distributional differences are displayed in the violin plots, displaying KDE (smoothed histograms) of each dataset. Grubb’s analysis was used to detect significant outliers $\left( {p < 0.05} \right)$ before displaying distributional differences in the violin plots of each dataset. Two outliers were detected, one belonging to the c1:g1 treatment group and one belonging to the c1:g3 treatment group. No significant outliers were detected in the c3:g1 treatment group. The outliers were excluded from the results before analyzing the distribution. It is apparent that the distribution of $\Delta E$ for the activity of c1:g3 is bimodal, while c1:g1 and c3:g1 are unimodal. Pairwise distributions of Young’s modulus $\left( E \right)$ between the control and each treatment group are also shown via violin plots (see Supplementary Fig. S1 online) and data can be found in Supplementary Table S1 online.

Both the mechanical properties, stiffness and peak load, were statistically analyzed. Statistical analysis by the Kruskal–Wallis test $\left( {p < 0.05} \right)$ showed that the stiffness as well as peak load at 20% strain resulting from the indentation tests on the c3:g1 treated cartilage were significantly different at the $\alpha =0.05$ level of significance. The stiffness and peak load results from both c1:g1 and c1:g3 groups showed no significant difference $\left( {ns} \right)$ following indentation testing. Dunn’s post hoc test for pairwise comparison of means was used to detect the difference between the control and treated group for all 3 treatment groups. Each treatment group was paired against established baseline control data to determine differences in peak load and stiffness after enzymatic degradation. At $\alpha =0.05$ level of significance, the c3:g1 treatment group shows significant difference in both peak load and stiffness when compared pairwise to its control indentation test results. The Dunn’s test showed no significant difference in peak load or Young’s modulus results from the c1:g1 or c1:g3 groups following enzymatic digestion. The degeneration of the mechanical integrity of cartilage is further validated with the histological analyses of the tissue in healthy and degraded states.

### Histological analysis

**Figure 4:**
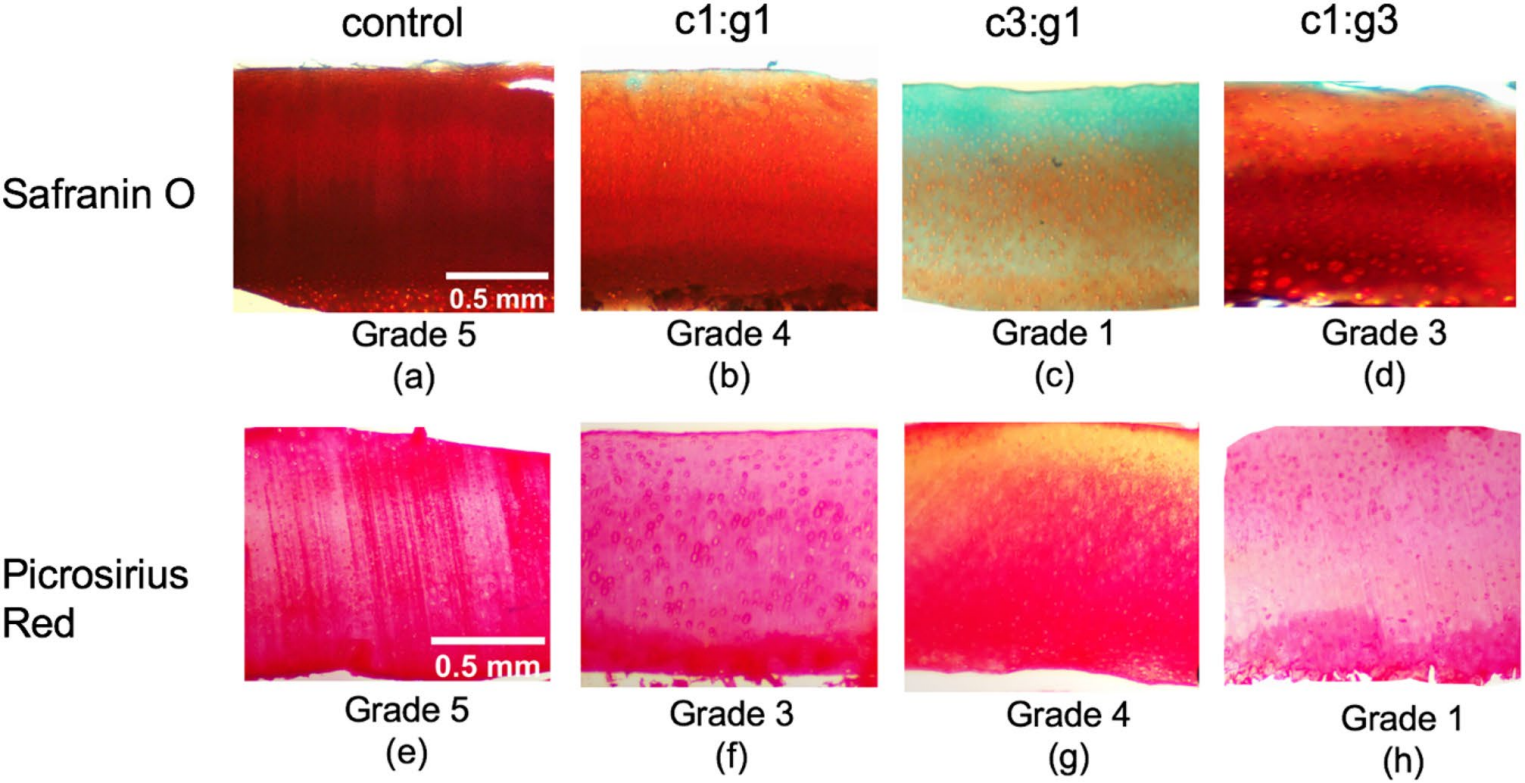
Change in intensity and consistency of the histochemical staining due to enzymatic activity compared to controls (**a**, **e**) in representative samples degraded with MMP-1 and MMP-9 of concentration c1:g1 (**b**, **f**), c3:g1 (**c**, **g**), and c1:g3 (**d**, **h**). Staining was performed with Safranin O to visualize PGs (**a**–**d**) and Picrosirius Red to visualize collagen (**e**–**h**). Classified as minimal (0), very weak (1), weak (2), moderate (3), strong (4), and very strong (5), scoring grade for each section is included for comparative analysis below the respective micrograph.

Representative images of Safranin O and Picrosirius Red stained cartilage sections are shown in Fig. 4. The untreated control group has strong Safranin O and Picrosirius Red staining indicating both normal collagen and PG content (Fig. 4a,e). Treatment groups c1:g1, c3:g1, and c1:g3 depict a weaker affinity for Safranin O uptake indicating a reduction in PG content starting at the articular surface (Fig. 4b–d), and reduced uptake in Picrosirius Red (Fig. 4f–h) compared to the control, indicating a reduction in collagen content as well. For Safranin O staining, the treatment group with the greatest abundance of collagenase, c3:g1 received the lowest grade by all three independent observers indicating minimal stain intensity. The qualitative (as well as comparative) assessment resulted in c1:g3 receiving a grade of 3, meaning independent observers believed the Safranin O staining intensity to be moderate, while the c1:g1 group received a grade of 4, indicating strong staining intensity. In these two groups, the intensity gradient is visible spanning from the superficial zone, where the treatment was applied, to the deep zone.

For Picrosirius Red, the three independent observers gave the c1:g3 group the lowest grade for staining intensity, whereas the c3:g1 group received a high grade of 4. The c1:g1 group received a moderate grade of 3 for staining intensity by the independent observers and exhibits a trace of staining intensity gradient from superficial to deep zone as well. Statistical distributions of the scoring grades are shown for all the treatment groups in the Supplementary section (see Supplementary Fig. S2 online). Individual grades given by each observer can be found in Supplementary Table S2 online.

**Figure 5:**
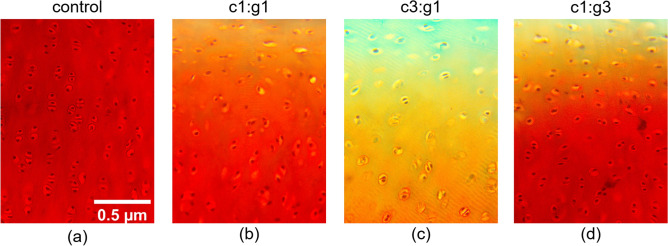
Higher magnification (40X) micrographs to demonstrate the change in intensity and consistency of the histochemical staining in the superficial zone due to enzymatic activity compared to controls (**a**) in representative samples degraded with MMP-1 and MMP-9 of concentrations c1:g1 (**b**), c3:g1 (**c**), and c1:g3 (**d**). Staining was performed with Safranin O to visualize PG content (**a**–**d**).

A close-up magnification of the cartilage superficial zone (40X) of Safranin O stained sections is displayed in Fig. 5 for both the untreated control (Fig. 5a) and the c1:g1, c3:g1, and c1:g3 treatment groups (Fig. 5b–d). When compared to the control, a degradation gradient can be seen in the treated samples indicating reduced proteoglycan content experienced by treated samples. The greatest reduction in Safranin O stain intensity in the superficial zone is shown in the c3:g1 group, indicating the greatest loss of proteoglycan content within this treatment group.

**Figure 6:**
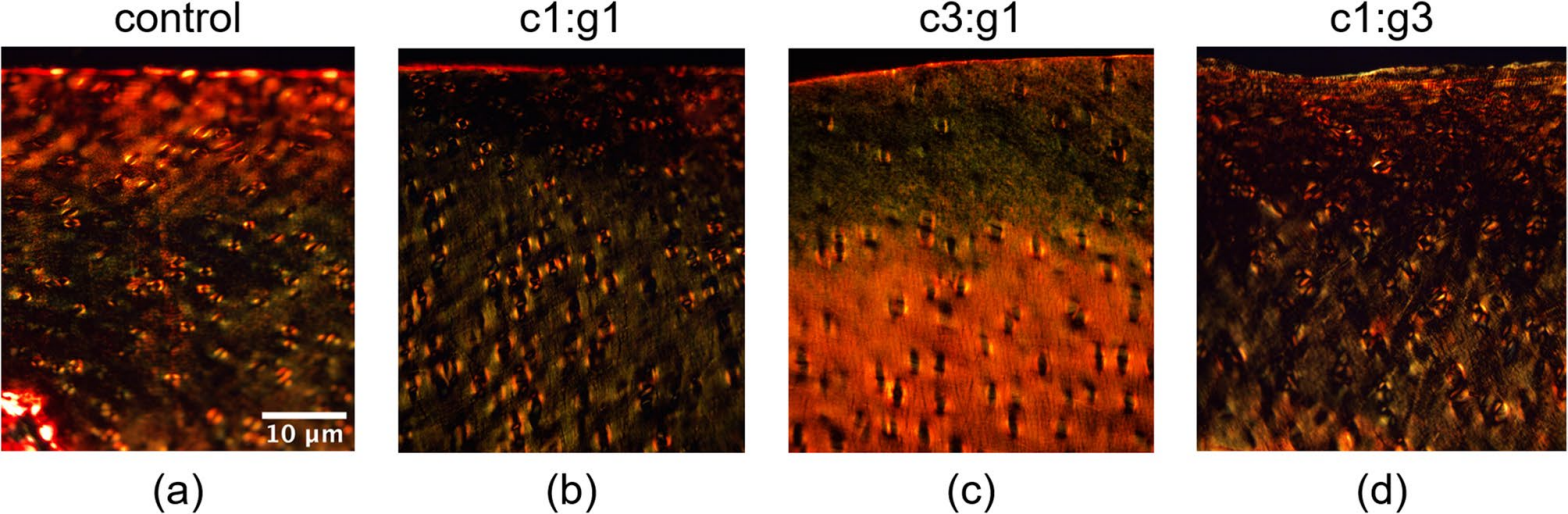
Collagen network visualization under polarized light microscopy of Picrosirius Red stained sections for untreated control (**a**), and MMP-1 and MMP-9 treatment groups c1:g1 (**b**), c3:g1 (**c**), and c1:g3 (**d**).

Picrosirius red staining highlights the natural birefringence of collagen when exposed to polarized light. Representative cartilage sections are shown in Fig. 6 for both control (Fig. 6a) and each enzymatic treatment group, c1:g1, c3:g1, and c1:g3 (Fig. 6b–d). Following degradation, collagen experiences content change and irregular arrangement causing the birefringence to exist in a different pattern than normal tissue such as the control (Fig. 6a). The c3:g1 treatment group (Fig. 6c) experienced the most irregular collagen arrangement highlighted by the weakened change in birefringence when compared to the control (Fig. 6a) indicating the greatest collagen degradation following enzymatic digestion by MMPs- 1 and 9. Furthermore, the Pearson correlation was used to assess the relationship between the loss in mechanical integrity and histological scoring. The resultant correlation coefficient of -0.96 implies that as loss of mechanical integrity increases, histological scoring decreases with the two instances being highly correlated.

## Discussion

Low levels of MMP expression are essential in regulating collagen homeostasis by digesting the collagen network; this balance is imperative for tissue development, remodeling, and repair^41^. This balance is disrupted either by mechanical stress or diseases such as OA, resulting in increased digestion of cartilage ECM constituents. All of the degradative enzymes belonging to the collagenase family (MMP-1, 8, and 13) are expressed at low levels in healthy joint tissue and experience increased expression in arthritic tissue. Characteristically, MMP-1 and 8 have been localized to the more superficial cartilage surface, while MMP-13 is found within the deeper layers^42,43^. The distribution pattern reflects the fact that MMP-1 and MMP-8 are primarily products of synovial cells and neutrophils, respectively, which are adjacent to the cartilage, while MMP-13 expression predominates in the chondrocytes^3^. Gelatinases are responsible for cleaving the proteoglycan core protein–aggrecan; however, enzymes included in the gelatinase family (MMPs- 2 and 9) further degrade collagen after collagenase has cleaved the collagen triple helix^3^. The mechanical integrity of the cartilage ECM is crucial in determining the tissue’s biomechanical properties, as the type II collagen and PG aggregates provide the cartilage with tensile and compressive strength^44^.

In the current study, we developed an in vitro cartilage degradation model by treating the articular surface with the two subfamilies of MMPs, MMP-1 and MMP-9 in different combinations. The MMPs in synergy were responsible for facilitating collagen and PG degradation in the cartilage ECM^45^. MMP-1 (c) and MMP-9 (g) have exhibited their destructive ability to denature and digest collagen fibrils at the molecular level^19^, whereas the current study aims to present their synergistic effect on cartilage tissue. Indentation tests were conducted in the current study as it is a classical technique to quantify the mechanical properties of articular cartilage in situ. Evidently, the mechanical integrity of the cartilage was compromised (Fig. 1) when treated with higher concentration of MMP-1, c3:g1 that degrades more collagen fibrils primarily at the superficial layer. Cartilage demonstrates the highest tensile stiffness at the superficial layer where fibrils are densely packed and parallel to the articular surface. However, PG aggregates occupy the interfibrillar space of the cartilage ECM and may act as a barrier to protect collagen fibrils from enzymatic denaturation^46^. From the indentation testing, it is inferred that the collagen fibers are primarily responsible for mechanical integrity not the PG aggregates, as we see a nearly similar variation for c1:g1 and c1:g3 (Fig. 1). The stress variation between the control and treated group at 20% bulk strain is nearly 24%, and 29% for c1:g1 and c1:g3, and ~ 50% for c3:g1. The increased concentration of MMP-9 (gelatinase) has little effect on aggregate degeneration. When the amount of collagenase is the more potent component in the enzyme mixture, combined with the presence of gelatinase, a much greater degradative impact is observed in the compressive cartilage stiffness (Fig. 2) as collagenase denatures the type II collagen in the ECM facilitating further digestion by gelatinase (MMP-9). The variability in stiffness changes $\left( {\Delta E} \right)$ for the concentrations of enzyme demonstrates a central tendency for c1:g1, where we observe a larger difference in the shape of the distribution from the other two (Fig. 3), signifying susceptibility of cartilage damage due to MMPs imbalance.

In the current study, histological analysis was used to supplement and validate the changes in macromechanical properties following enzymatic degradation observed through indentation testing. From the histological analysis, it is apparent that the greatest visibility of fast-green counterstain lies within the articular surface of the c3:g1 group (Fig. 4c). The counterstain presence is indicative of depleted proteoglycan content as there is little to no uptake of Safranin O at the surface. Furthermore, the intense staining affinity for Picrosirius Red at the deep zone with yellow at the surface (Fig. 4g) is the evidence of reduced collagen content at the surface and normal collagen content in the middle and deep zones. The bulk of visible degradation for all groups is occurring within the superficial layer of the articular surface, the magnified view of this zone (Fig. 5) provides an enhanced view of the highly reduced Safranin O uptake from the c3:g1 group (Fig. 5c) which indicates proteoglycan degradation. It is apparent that the collagen cleavage by MMP-1, reduces the tissue level integrity, which enables the penetration of more MMP-9, and thereby degrading more PG aggregates. Additionally, the collagen content change and irregular configuration observed in the birefringence of the Picrosirius Red stained c3:g1 group (Fig. 6c) when compared to the respective control (Fig. 6a) captured through polarized microscopy provides further evidence of collagen degradation. From the results, we have concluded that the c3:g1 group experienced the greatest loss of both PGs and collagen at the articular surface, indicated by the abundance of fast green and picric acid present in the superficial zone of the c3:g1 treated samples. In healthy tissues, aggregating proteoglycans that are occupying the interfibrillar zone may act as a barrier to protect the cross-linked collagen fibrils from enzymatic denaturation^46^. With the aggregating proteoglycan barrier compromised by the presence of MMP-9, collagenase receives access to the type II collagen fibrils paving the way for further degradation of denatured collagen by gelatinase. Subsequently, samples treated with the increased concentration of collagenase (c3:g1) experience the greatest degradative force once the PG barrier is compromised and the concentration of collagenase is increased. The degradation of cartilage appears largely dependent on enzyme diffusivity, which shows no or minimal diffusivity beyond the superficial layer. The collagenolytic activity is primarily increased by the abundance of MMP-1 and aggravated by MMP-9 in addition to its gelatinolytic activity. The reduced PG content exhibited by light orange intensity at the surface of the other two enzyme concentrations (Fig. 5b,d) shows the gelatinolytic activity of MMP-9 with different concentration. The histochemical staining delineates that the abundance of MMP-1 plays an important role in collagen degradation and is aggravated by MMP-9.

Exploring the effect of degraded fibrils on cartilage mechanics is experimentally challenging. While the results from this study provide promising insight into how the relative abundance of enzymes MMPs-1 and 9 may impact the biomechanical response of articular cartilage, the differences in the control states of the samples from multiple bovines may introduce variance in the results. Samples are separated by bovine within their respective treatment groups, but samples from both medial and lateral condyles belonging to the same bovine were mixed and randomized based on a mechanical characterization study finding no significant difference in the frictional coefficient between the two condyles^47^. While the age of all bovines utilized in this study was similar (~ 2 years in age), all the specimens might not be an exact age match and potential pre-existing conditions of the subjects were also unknown which could attribute to the differences in control states between treatment groups. However, a thorough visual check was performed to identify any injury or disease state, and concomitant thickness measurement showed no large variations, indicating a healthy state among the joints. Rather than enzymes found in vivo^34^, prior studies used bacterial collagenase that contains small amounts of proteases, which can degrade the proteoglycans within the ECM^34,48^. Moreover, mammalian MMP-1 cleaves collagen at a single site in each chain of the triple helix, whereas bacterial collagenase initially cleaves all three chains at multiple domains along the triple helix, followed by continued cleavage of the collagen fragments into multiple smaller fragments.

In summary, MMP mediated articular cartilage degradation is closely tied with trauma or physiological conditions and is widely accepted as fundamental to OA disease progression. However, the mechanistic investigation of the synergistic influence of MMPs of a different kind (i.e., collagenase and gelatinase) is limited. The results of this study illuminate in vitro the degradative effect of MMPs-1 and 9 on articular cartilage biomechanical properties. From the results, it can be concluded that combined collagenase and gelatinase weaken the compressive strength of the cartilage extracellular matrix, but a much greater destructive force is observed when collagenase is present in increased concentration. Cartilage, being avascular, has a limited capacity for intrinsic healing and repair. Preservation of articular cartilage is essential to joint-health and is highly dependent on maintaining the integrity of the tissue’s internal organized architecture. This insight into the combined degradative influence of MMPs on articular cartilage will aid in research developing targeted MMP inhibition and therapeutic interventions to prevent cartilage destruction as well as early-stage OA.

## Materials and methods

Articular cartilage plugs tested in this study were harvested from 3 different fresh stifle joints of mature cows of approximately ~ 2 years old, collected from a local slaughterhouse. The bovine stifle joints were stored frozen at -50˚C until the time of sample (plug) extraction. Prior studies have shown that freeze–thaw cycles have negligible impact on the mechanical properties of articular cartilage explants^49,50^.

### Sample preparation

**Figure 7:**
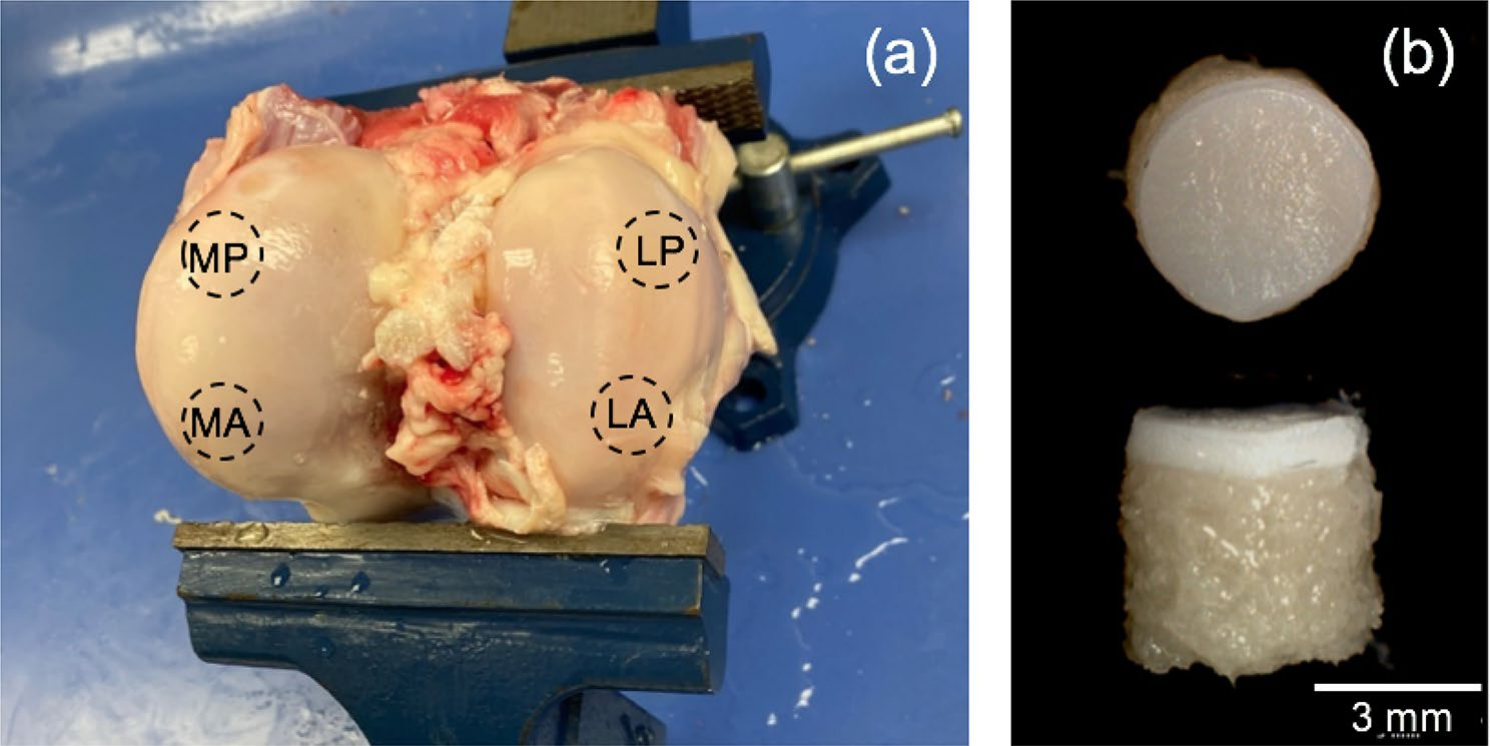
(**a**) Femoral condyle locations used for sample extraction. Medial posterior (MP), medial anterior (MA), lateral posterior (LP), and lateral anterior (LA) sample extraction locations are denoted. (**b**) Extracted articular cartilage plug with subchondral bone.

The stifle joints were dissected at room temperature to expose the medial and lateral femoral condyles from where the samples were extracted^47^. A total of 30 articular cartilage plugs of diameter 3.5 mm and 5 mm thickness with subchondral bone attached were harvested from the femoral condyles of the 3 stifle joints (Fig. 7) using a mosaicplasty tubular chisel (Smith & Nephew 7,209,234)*.* During the extraction process, the articular surface was moistened with phosphate-buffered saline (PBS) to prevent the cartilage from drying out. Cartilage quality was judged before and after the extraction by visible inspection, and cartilage showing any sign of fissures or surface defects were excluded from the current study. The harvested samples were stored frozen at -20˚C in PBS moistened gauze until needed for testing^51^. Prior to testing, cartilage thickness (without subchondral bone) was individually assessed using a needle probe of the micromechanical tester, Mach-1 v500c (Biomomentum Inc.). The average thickness of the articular cartilage was 1.3 ± 0.08 mm.

### Enzymatic degradation of articular cartilage

To prepare MMPs for experimental use, vials containing MMPs were tapped down to ensure all of the lyophilized powder was collected at the bottom of the vial prior to opening. Per the manufacturer’s instructions (BioVision) MMPs were reconstituted in PBS solution at a concentration of 0.5 mg/mL and allowed to sit for 30 min. Vials containing MMPs were gently agitated during the reconstitution process prior to making dilutions. No vigorous shaking was induced as this can cause foaming and protein denaturation. Following dilutions, 200 µL aliquots were stored at -20˚C until needed for enzymatic degradation. To induce downward degradation from the articular surface, the explants were individually placed inside the wells of a standard well plate. A mixture of 2% agarose gel was prepared using solid agar (Sigma-Aldrich) and TAE buffer (Sigma-Aldrich). Warm agarose was then carefully pipetted around each sample until only the top of the articular surface was exposed to the enzyme solution to simulate in vivo conditions. The agarose gel was allowed to cool and solidify for 30 min before the top articular surface of each sample was loaded with activated human recombinant MMP-1 and MMP-9 (BioVision, Milpitas, CA) mixtures with different concentrations. In groups of 10, articular cartilage samples were treated with a MMP-1(c) to MMP-9 (g) ratio as shown in Table 1. The concentration scale of MMPs used for sample degradation was selected based on studies utilizing immunoassays (ELISA) to detect the pro-form of the enzymes in the synovial fluid of both osteoarthritis and inflammatory arthritis patients. The level of proMMP-1 detected in the synovial fluid of arthritis patients did not exceed 6 ng/mL in trials including over 300 patients^52^. Thus, this study was also sought to mimic a similar enzymatic degradation experienced by articular cartilage during early-stage OA.

**Table 1:** Concentration ratios of enzymes used to degrade articular cartilage samples.

MMP-1 (c) concentration (ng/mL)	MMP-9 (g) concentration (ng/mL)	c:g ratio
4	4	1:1
2	6	1:3
6	2	3:1

With a closed lid, the well plate containing the enzymatically treated samples was incubated at 37˚C, 5% CO_2_ for 44 h^39,53^. Every 12 h, the samples were gently agitated to promote diffusion. Upon completing the incubation period, explants were thoroughly washed (3 × for 10 min) and rinsed in PBS solution before proceeding to indentation testing.

### Indentation testing

Each sample was placed inside an osteochondral core holder to secure the sample during the testing process and prevent displacement. The sample was immersed in a PBS bath throughout testing to prevent drying of the articular surface. Each articular cartilage explant was subjected to compressive stress-relaxation tests using a spherical indenter (1 mm dia.) in the Mach-1 testing apparatus, which is a gold standard for cartilage mechanical testing. The Mach-1 micromechanical tester is non-destructive, accurately measures tissue-level properties, and permits the use of multiple indenter geometries. Both flat ended and spherical indenters have been used for indentation testing^31,32^. However, the spherical type of indenter was chosen for the experiment as it causes less damage to the cartilage compared to pyramid and other sharp indenter tips. A spherical indenter has been shown to create more centralized compression in the tissue and less stretching at the edges as compared to a flat indenter. Additionally, the spherical indenter deforms a more narrow and shallow region of the cartilage^31^. Spherical indenter size (1 mm in diameter) was selected based on a literature review examining techniques used to mechanically assess cartilage samples. The results of the review determined most studies aiming to mechanically characterize articular cartilage use flat indenters for unconfined/confined compression tests, while in situ tests may opt for spherical or flat indenters with around 30% of studies not specifying indenter geometry^54^. It is accepted that in order to ensure full contact between the probe and the sample, the sample diameter must be greater than that of the probe. Prior test results showed that both 1 mm as well as 3 mm indenter tips were significantly better when compared to the 0.5 mm indenter with a standard deviation of about 10% of the average^55^. Furthermore, the computational study by Meloni, G.R., et al.^56^ showed that at minimum the probe to sample diameter ratio must be 1:3 to accurately measure reaction forces and fluid pressurization^56^. In the current study, this ratio is 1:3.5 which is beyond the threshold dictated by the prior in silico analysis. Samples were compressed at a strain rate of 0.01%/sec until the total strain reached 20%^54^, which typically falls within the elastic limit. The test was repeated on the same sample, allowing the samples to relax for 2 h between the tests^39^. To ensure repeatability, the percent change in sample results between control tests was monitored. After the resting period, sample test results experienced a negligible amount of change (≤ 2%). Each sample was tested in a healthy state prior to introducing degradative enzymes MMP-1 and MMP-9 mixture to establish a baseline. After enzymatic treatment, the same tests were conducted twice for each sample (i.e., 4 tests total per sample), allowing each sample to serve as its own control. To investigate the differences in mechanical properties between healthy and degraded states, the peak load $\left( F \right)$ required to strain the sample and Young’s modulus $\left( E \right)$ were determined from the indentation test.

### Histological analysis

Both healthy and enzymatically degraded specimens were fixed at 4˚C in 10% phosphate-buffered formalin (Sigma-Aldrich) for 24 h. Since decalcifying agents such as EDTA, nitric and hydrochloric acids have shown the potential to extract or destroy proteoglycans present in cartilage resulting in diminished binding of Safranin O/Fast Green stains^57,58^, cartilage was carefully removed from the subchondral bone prior to histological preparation. Following fixation, cartilage samples were thoroughly rinsed in running tap water for 10 min, then dehydrated through a series of graded ethanol changes from 50%-100%. Samples were then cleared with xylene before being embedded in Paraplast Plus paraffin (Electron Microscopy Sciences) and sectioned with a rotary microtome. Sections were mounted on plain glass slides for histological analysis. 12 μm thick sections were deparaffinized and rehydrated for either Safranin-O/Fast Green (Sigma-Aldrich) or Picrosirius Red (Sigma-Aldrich) staining using standard protocols^58^. The intensity of the Safranin-O stain is proportional to the proteoglycan content present in the articular cartilage samples, while the Picrosirius Red intensity is proportional to the collagen content. The reduction of the histochemical staining in the degraded samples will confirm the loss in PG and collagen content compared to controls, thus suggesting effective enzymatic degradation of the cartilage.

Micrographs of both controls and degraded stained samples were obtained using AmScope biological microscope XSG Series under 10X magnification to observe the degradation. For a magnified close-up view of the superficial zone 40X magnification was used. Polarized light microscopy images were taken using a Leica DMLP polarizing light microscope to observe collagen content and arrangement. Qualitative analysis of both Safranin-O and Picrosirius Red sections were graded on a scale of 0 to 5 by three independent observers where the stain intensity is classified as minimal (0), very weak (1), weak (2), moderate (3), strong (4), and very strong (5)^59^.

### Statistical analysis

Statistical analyses were performed using OriginPro 2019b (OriginLab Corporation, Northampton, MA). Numerical data are presented as *mean* ± *SD* and graphical results are *mean* with standard deviation (*SD*) as error bars unless otherwise stated. Due to the relatively small number of samples and nonnormality of the data, the nonparametric Kruskal–Wallis test followed by Dunn’s post hoc test for pairwise comparison was used to assess statistical differences^60^ of mechanical properties between pre-selected pairs (i.e., control vs. treated for each ratio of enzymes). The histological scoring was also expressed in $mean \pm SD$. To assess the relationship between histological scoring and loss of mechanical integrity the Pearson Correlation was used in the current study. A significance level was set at $p < 0.05$ and was employed for all tests.

## Supplementary Information

Supplementary Information.
