# Supplementary material for: An in vitro investigation to understand the synergistic role of MMPs-1 and 9 on articular cartilage biomechanical properties
Source: Sci Rep. 2021 Jul 13;11:14409. doi: 10.1038/s41598-021-93744-1 (PMC8277889; doi:10.1038/s41598-021-93744-1)
Supplement: Supplementary file 1 — Supplementary Information. [file 41598_2021_93744_MOESM1_ESM.docx]

**Supplementary Information**


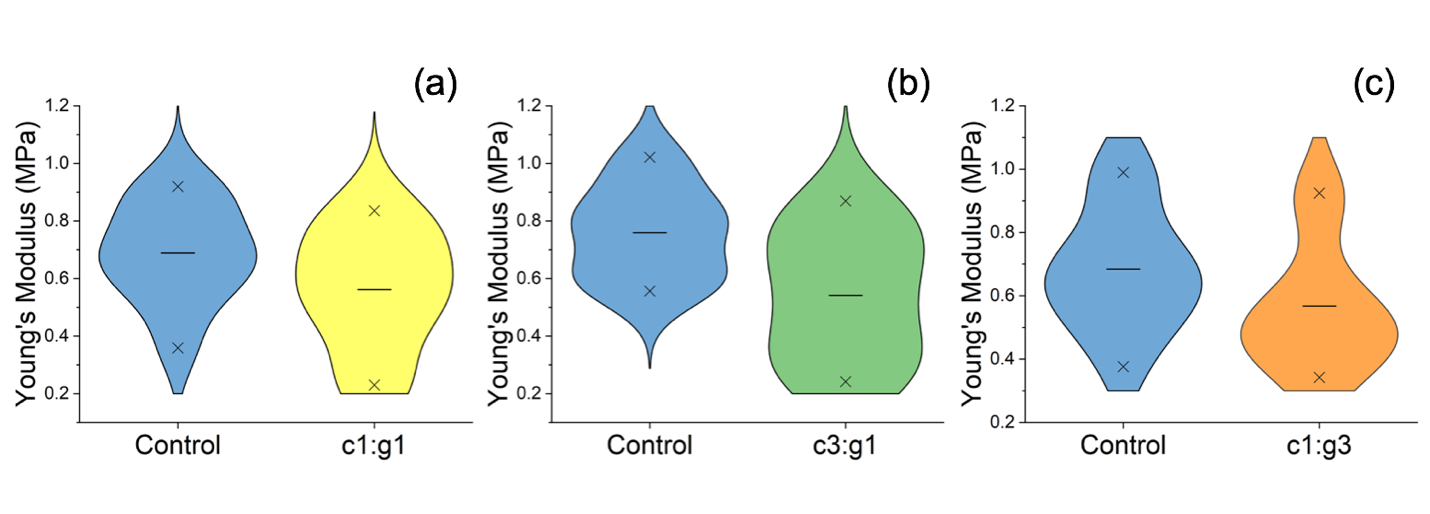


**Supplementary Figure S1.** Distributions of Young’s Modulus $\left(E\right)$ prior to and following enzymatic digestion by MMPs- 1 and 9 for the c1:g1 (a), c3:g1 (b), and c1:g3 (c) treatment groups. The mean from each dataset is designated by a straight line while the 99^th^ (top) and 1^st^ (bottom)percentiles are indicated by crosses within each plot.

**Supplementary Table S1.** Young’s modulus data generated through indentation testing for each group (*n* =10) prior to and following enzymatic digestion by MMPs-1 and 9 for the c1:g1, c3:g1, and c1:g3 treatment groups. Changes in the raw values of Young’s Modulus $(E)$ are expressed as $(\Delta E)$.

| **Sample Name** | **Treatment Group** | **Control YM (MPa)** | **Treated YM (MPa)** | **Δ E** |
| --- | --- | --- | --- | --- |
| A1 | c1:g1 | 0.630 | 0.483 | 0.147 |
| B1 | c1:g1 | 0.920 | 0.836 | 0.084 |
| C1 | c1:g1 | 0.841 | 0.757 | 0.084 |
| D1 | c1:g1 | 0.696 | 0.230 | 0.466 |
| E1 | c1:g1 | 0.359 | 0.247 | 0.112 |
| F1 | c1:g1 | 0.594 | 0.557 | 0.037 |
| A2 | c1:g1 | 0.744 | 0.656 | 0.088 |
| B2 | c1:g1 | 0.901 | 0.790 | 0.112 |
| C2 | c1:g1 | 0.517 | 0.460 | 0.057 |
| D2 | c1:g1 | 0.682 | 0.601 | 0.081 |
| E2 | c3:g1 | 0.618 | 0.359 | 0.259 |
| F2 | c3:g1 | 0.602 | 0.366 | 0.235 |
| A3 | c3:g1 | 0.788 | 0.552 | 0.235 |
| B3 | c3:g1 | 0.557 | 0.246 | 0.311 |
| C3 | c3:g1 | 0.601 | 0.242 | 0.359 |
| E3 | c3:g1 | 0.829 | 0.697 | 0.132 |
| F3 | c3:g1 | 1.021 | 0.870 | 0.152 |
| A4 | c3:g1 | 0.833 | 0.735 | 0.098 |
| B4 | c3:g1 | 0.974 | 0.802 | 0.172 |
| C4 | c3:g1 | 0.770 | 0.542 | 0.228 |
| D4 | c1:g3 | 0.989 | 0.924 | 0.065 |
| E4 | c1:g3 | 0.704 | 0.632 | 0.072 |
| F4 | c1:g3 | 0.656 | 0.502 | 0.154 |
| A5 | c1:g3 | 0.975 | 0.904 | 0.071 |
| B5 | c1:g3 | 0.789 | 0.491 | 0.298 |
| C5 | c1:g3 | 0.376 | 0.342 | 0.034 |
| D5 | c1:g3 | 0.585 | 0.415 | 0.170 |
| E5 | c1:g3 | 0.693 | 0.557 | 0.137 |
| F5 | c1:g3 | 0.507 | 0.424 | 0.083 |
| A6 | c1:g3 | 0.567 | 0.485 | 0.082 |

**
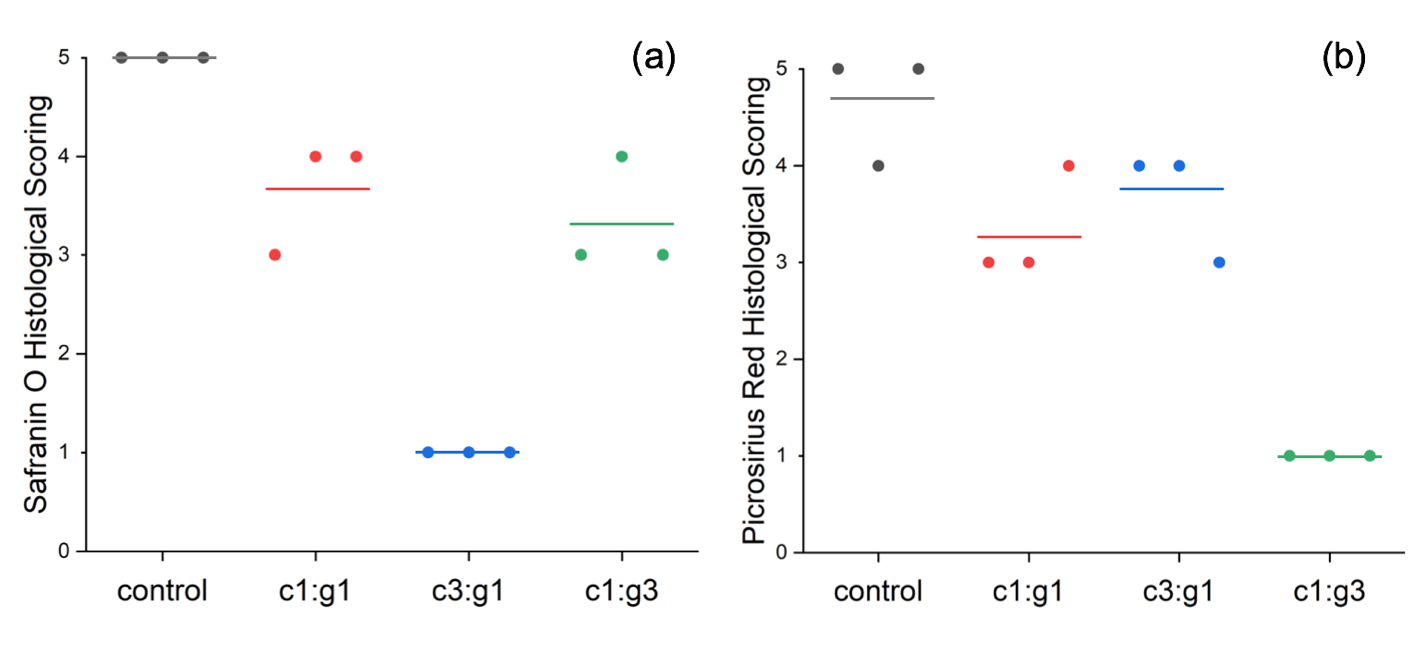
**

**Supplementary Figure S2.** Distributions of histological scoring grades for Safranin O (a) and Picrosirius Red (b) stained cartilage sections generated by three independent observers used for qualitative analysis. Sections were graded on a scale of 0 to 5 by three independent observers where the stain intensity is classified as minimal (0), very weak (1), weak (2), moderate (3), strong (4), and very strong (5). Each dot represents the grade selected by a single observer for the respective treatment group while the mean value is represented by a straight line.

**Supplementary Table S2.** Scoring grades given by each individual independent observer for Safranin O and Picrosirius Red stained cartilage sections graded on a scale of 0 to 5 where the stain intensity is classified as minimal (0), very weak (1), weak (2), moderate (3), strong (4), and very strong (5).

| Independent Observer | Control  Safranin O Intensity Rating (0-5) | c1:g1  Safranin O Intensity Rating (0-5) | c3:g1  Safranin O Intensity Rating (0-5) | c1:g3  Safranin O Intensity Rating (0-5) |
| --- | --- | --- | --- | --- |
| 1 | 5 | 3 | 1 | 3 |
| 2 | 5 | 4 | 1 | 4 |
| 3 | 5 | 4 | 1 | 3 |
| Mean ± SD | 5 ± 0 | 3.67 ± 0.58 | 1 ± 0 | 3.33 ± 0.58 |
| Independent Observer | Control  Pic. Red  Intensity Rating (0-5) | c1:g1  Pic. Red  Intensity Rating (0-5) | c3:g1  Pic. Red  Intensity Rating (0-5) | c1:g3  Pic. Red  Intensity Rating (0-5) |
| 1 | 5 | 3 | 4 | 1 |
| 2 | 4 | 3 | 4 | 1 |
| 3 | 5 | 4 | 3 | 1 |
| Mean ± SD | 4.67 ± 0.58 | 3.33 ± 0.58 | 3.67 ± 0 | 1 ± 0 |
